# Oral Feeding of Cow Milk Containing A1 Variant of β Casein Induces Pulmonary Inflammation in Male Balb/c Mice

**DOI:** 10.1038/s41598-020-64997-z

**Published:** 2020-05-15

**Authors:** Shikha Yadav, Nakul Dev S. Yadav, Atish Gheware, Ankur Kulshreshtha, Pankaj Sharma, V. P. Singh

**Affiliations:** 1 Amity Institute of Biotechnology, Amity University, Uttar Pradesh Sector-125, Noida, 201313 India; 2 National Institute of Biologicals, Plot No. A-32, Sector-62 Institutional Area, Noida, 201309 India; 3 District Disease Diagnostic Laboratory, Nuh, Department of Animal Husbandry & Dairying, Haryana, 122107 India; 4 CSIR-Institute of Genomics & Integrative Biology, Mathura Road, New Delhi, 110025 India

**Keywords:** Metabolomics, Respiration, Asthma, Experimental models of disease

## Abstract

Milk is globally consumed as a rich source of protein and calcium. A major protein component of milk is casein, with β-casein having 2 major variants A1 and A2. Of these, A1 casein variant has been implicated as a potential etiological factor in several pathologies, but direct effect on lungs has not been studied. The objective of the present study was to evaluate the A1and A2 β casein variants of cow milk as factors causing allergic airway disease in murine model. Mice fed with A1A1 milk exhibited increased airway hyperresponsiveness with increasing concentration of bronchoconstrictor (methacholine), which was not observed in mice fed with A2A2 milk. Significantly elevated levels of IL-4 and IL-5 were found in bronchoalveolar lavage and serum of A1A1 variant fed mice. Increased IgE and IgG levels along with increased infiltration of lymphocytes and eosinophils, leading to peribronchial inflammation was also observed in A1A1 variant fed mice, although, no goblet cell hyperplasia or airway remodeling was observed. In contrast, A2A2 milk fed mice presented phenotype matching the control group, while A1A2 milk fed group presented an intermediate phenotype. In summary, our results show that A1 form of cow milk has a proinflammatory effect on the lung resulting in phenotype closely matching with the typical allergic asthma phenotype.

## Introduction

Milk is consumed worldwide by infants, children and adults as a high quality source of protein and calcium. As per Food and Agriculture Organization (FAO), cows accounted for about 82% of 828 million tonnes of total milk produced globally in 2017. Caseins account for approximately 78–82% of total milk protein^1^. Of the different casein forms, β-casein has 13 variants in bovine milk, of which A1 and A2 are the most common variants, differing only at position 67, which is histidine in A1 and proline in A2 milk. It has been suggested that the first domesticated cows contained the A2 variant of β-casein^2^ including Indian cattle^3^. During evolution, the European cattle had a mutation of P67H i.e proline at 67^th^ position was replaced with histidine; hence while the American and European breeds such as Holstein–Friesian mainly contain A1 β-casein, the Old World Guernsey and Jersey breeds and Indian cattle contain A2 β-casein^2^. Apart from proteins and minerals, bioactive peptides released from milk proteins have also been demonstrated to have beneficial health effects^4^. However, the presence of histidine at position 67 in the A1 β-casein variant results in a weak bond with the preceding isoleucine, making it susceptible to proteolytic cleavage resulting in release of opioid peptides named β-casomorphins (BCMs) of varying lengths, of which the seven- amino acid peptide is the most common^5^. While the milk containing the original A2 variant of β-casein has been found to be beneficial for human health, the A1 variant has been implicated as a potential etiological factor in a number of human diseases including ischemic heart disease, diabetes mellitus-1 and autism^2,6–11^. As milk is consumed orally and digested in the gut, most of the studies have focused on the gastrointestinal effects of A1/A2 forms of β-casein^12^. A recent study demonstrated that consumption of milk containing A1 β-casein worsened gastrointestinal symptoms^13^. In a mouse model, feeding of β-casein from A1/A1 and A1/A2 cows increased Th2-driven gut inflammation^14^ and similar results were obtained by gavage of BCM-5 or BCM-7.

Similar to its effects on gut inflammation, cow milk has been found to have immunomodulatory effects on respiratory health^15^. Feeding of mice with raw cow milk prevented airway inflammation in house dust mite-induced asthma model while processed milk did not; probably due to loss of some heat-labile components^16^. As both pulmonary and gut inflammation are very similar, being Th2-driven and involving deregulation of mucosal immunity, it has been suggested that a subgroup of individuals with respiratory ailments involving mucus overproduction may benefit from elimination of dairy components from their diet^17^, though a systemic investigation into underlying mechanisms hasn’t been performed yet. Therefore, we have undertaken this study to evaluate the A1 and A2 β casein variants of cow milk as factors causing allergic airway disease in murine model. Long-term feeding of mice with A1A1 milk for around 30 weeks significantly increased methacholine induced airway hyperresponsiveness and was accompanied by increased recruitment of lymphocytes, eosinophils and total inflammatory cells in both bronchoalveolar lavage (BAL) and blood. It was primarily a Th2 response, as suggested by increase in the Th2 cytokines i.e IL-4 and IL-5 with no significant difference in the levels of IFNγ which is Th1 cytokine; along with increased IgE in both BAL and serum and IgG levels in BAL. Interestingly, while A2A2 milk had no negative effects, with trends instead suggesting a protective role if any, feeding the mice with A1A2 milk produced intermediate inflammatory effects.

## Materials and Methods

### Genotyping of cows

Holstein Friesian and Hariana cows were genotyped to screen for A1 and A2 variants of β casein to obtain A1A1, A1A2 and A2A2 combinations of milk for the study. Blood samples were collected in sterile tubes containing EDTA as anticoagulant. Genomic DNA was isolated by using Ultra clean BloodSpin DNA Isolation Kit (MOBIO) and purity and concentration of DNA was established using nanodrop method (Thermo Scientific Equipment). Allele Specific PCR was carried out on Agilent Technologies Sure Cycler 8800 as described earlier^18^ using 50 ng of genomic DNA in a final reaction volume of 10 µl. The PCR conditions were: initial denaturation at 95 °C for 5 min, followed by 30 cycles of 95 °C for 30 sec, 65 °C for 45 sec and 72 °C for 45 sec and a final extension of 72 °C for 10 min. PCR products were visualized following electrophoretic separation in 2.0% agarose gel.

### Milk collection

A1A1, A1A2 and A2A2 combinations of milk were collected from these genotyped dairy animals after they were screened negative for mastitis. The milk samples were transported at 2–8 °C, boiled at 100 °C for 2 minutes and after cooling to room temperature were stored overnight at 4–6 °C. The fat was then removed and milk was used for feeding the Balb/c mice under present investigation. Fresh milk was collected from genotyped cows every 4–5 days.

### Animals

Specific Pathogen Free (SPF) male BALB/c mice (3–4 weeks old) were obtained from National Institute of Biologicals (NIB), Noida, Uttar Pradesh, India and transported and maintained at CSIR- Institute of Genomics & Integrative Biology (IGIB), Mathura Road, New Delhi, India where they were housed in Individually Ventilated Cages (Citizen) with corncob as bedding and 12:12 hr day and night cycle. They were fed balanced pellet diet (Golden Feed, Delhi). All animals were housed at a temperature of 20–25 °C, relative humidity between 50–60% and were maintained according to guidelines of Committee for the Purpose of Control and Supervision of Experiments on Animals (CPCSEA). The study was designed and performed in accordance with the approvals granted by the Institutional Animal Ethics Committee (IAEC) of CSIR-IGIB, India.

Mice were randomly divided into 4 experimental groups which were named according to the treatment given for a period of 30 weeks i.e. Control, A1A1, A1A2 and A2A2, receiving RO water (water purified by reverse osmosis), A1A1, A1A2 and A2A2 β casein variants of milk respectively at a dose rate of 10 ml/kg body weight, 5 days/week by oral gavage, in addition to the standard pellet diet.

### Measurement of airway hyperresponsiveness (AHR)

Airway function of mice was measured as enhanced pause (Penh) using whole body plethysmography (Buxco Electronics, USA) as described^19^. Each mouse was housed in the whole body plethysmograph and acclimatized. Basal Penh levels were obtained by using 100ul of PBS as aerosol. Subsequently, aerosol of increasing concentrations of methacholine (0–60 mg/ml) purchased from Sigma (USA) was given for 60 seconds each and Penh values were obtained. The aerosol was generated using ultrasonic nebulizer (Aeronob Pro, Buxco). After a gap of one week, airway resistance was estimated using the FlexiVent system (SCIREQ, Montreal, Canada), which integrates the computer-controlled mouse ventilator with the measurements of respiratory mechanics as described previously^20^ in tracheostomized mice under anesthesia (Xylazine 10 mg/kg and Thiopentone 60 mg/kg, intraperitoneal). The results were expressed as enhanced pause (Penh) and airway resistance (fold change) with increasing concentrations of methacholine.

### Collection of bronchoalveolar lavage (BAL) fluid and blood samples

Immediately after measurement of airway resistance by FlexiVent, mice were euthanized using an overdose of thiopentone sodium. Blood samples were collected via heart puncture; serum was separated by centrifugation at 3,000 rpm for 10 min and stored at −80 °C until use. The trachea was cannulated and BAL fluid was collected by washing lungs with 1.0 ml of PBS from each mouse which was then centrifuged (400 *g*, 4 °C, 10 min). The pellet was resuspended in PBS and used for cell count; the supernatant was stored at −80 °C until use.

### Cell count and lung histology

The BAL cells were washed three times with PBS and the pellet was resuspended in 200 ul of cold PBS. A small portion of this suspension was taken for total cell counts using Neubauer’s chamber. For differential counts, cytopsin preparations were made and stained with Leishman’s stain. The cells were identified and counted by standard morphology; 200 cells were counted and the absolute number of lymphocytes and eosinophils was calculated.

For lung histology, the lungs were excised and fixed in 10% buffered formalin. The fixed, paraffin embedded tissues were cut into 5um sections and stained with Hematoxylin and Eosin (H&E), Periodic Acid-Schiff (PAS) to assess inflammation and mucus secretion and Mason Trichrome (MT) for lung tissue remodeling and collagenisation respectively. Histopathological analysis was done in 4 mice from each group.

### Measurement of cytokine and immunoglobulin levels

The levels of cytokines IL-4, IL-5 and IFNγ, as well as IgG and IgE levels were measured in duplicates in serum and BAL fluid supernatant by sandwich ELISA (RayBiotech Inc, USA), as per manufacturer’s protocol.

### Statistical analysis

Results are presented as mean ± standard error of mean (SEM) and analysed by one-way ANOVA. Significance has been denoted by *P ≤ 0.05, **P ≤ 0.01, ***P ≤ 0.001 and ****P ≤ 0.000 using GraphPad prism.

## Results

### Determination of A1 & A2 β casein variants by Allele Specific PCR

A total of 15 cows were individually identified by ear tags, including 9 Holstein Friesian (HF) and 6 Hariana cows from organized farms. They were then genotyped using allele-specific PCR to obtain all the three β casein variants of cow milk i.e. A1A1, A1A2 and A2A2 (Table 1**)**. Two HF cows were found to have A1A1 β casein while another two had A2A2 form; the remaining 5 HF cows had A1A2 variant of β casein. All the 6 Hariana cows were found to have A2A2 variant of β casein.These genotyped cows were negative for mastitis and were used as source of milk for feeding of the mice.

**Table 1:** Screening of Cows for A1A1, A1A2 and A2A2 genotyes using allele-specific PCR.

S. No.	Breed of Cow	No of Cows Genotyped	Genotype
1.	Holstein Friesian	9	A1A1-2 cows A1A2-5 cows A2A2-2 cows
2.	Hariana	6	A2A2-6 cows

### A1A1 β-casein variant of cow milk increases airway hyperresponsiveness in mice

**Figure 1:**
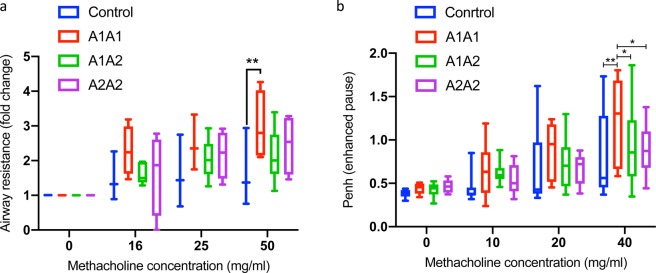
A1A1 milk exacerbates methacholine-induced airway hyperresponsiveness as measured by (**a**) Airway Resistance and (**b**) Penh (enhanced pause) values at different doses of methacholine after 30 weeks of feeding A1A1, A1A2 and A2A2 milk. Results are presented as mean ± standard error of mean (SEM) and analyzed by one-way ANOVA. n = 8–10 mice/group. Significance denoted by *P ≤ 0.05, **P ≤ 0.01, ***P ≤ 0.001 and ****P ≤ 0.000.

To determine the possible effects of A1A1, A1A2 & A2A2 β**-**casein variants on airway function, mice were fed with these variants at a dose of 10 ml/kg by oral gavage for 30 weeks and whole body plethysmography as well as FlexiVent was performed. We found that mice fed with A1A1 milk exhibited significantly higher Penh as compared to those fed with A2A2 milk (Fig. 1b). In addition, A1A1 milk feeding also induced airway resistance which was significantly higher as compared to control mice and those fed with A2A2 β**-**casein milk variant (Fig. 1a). Interestingly, mice fed with A1A2 milk exhibited airway response intermediate to mice fed with A1A1 or A2A2 milk, measured either by Penh (Fig. 1b) or airway resistance (Fig. 1a).

### A1A1 β-casein variant of cow milk increases Th2 cytokines levels in mice lungs

**Figure 2:**
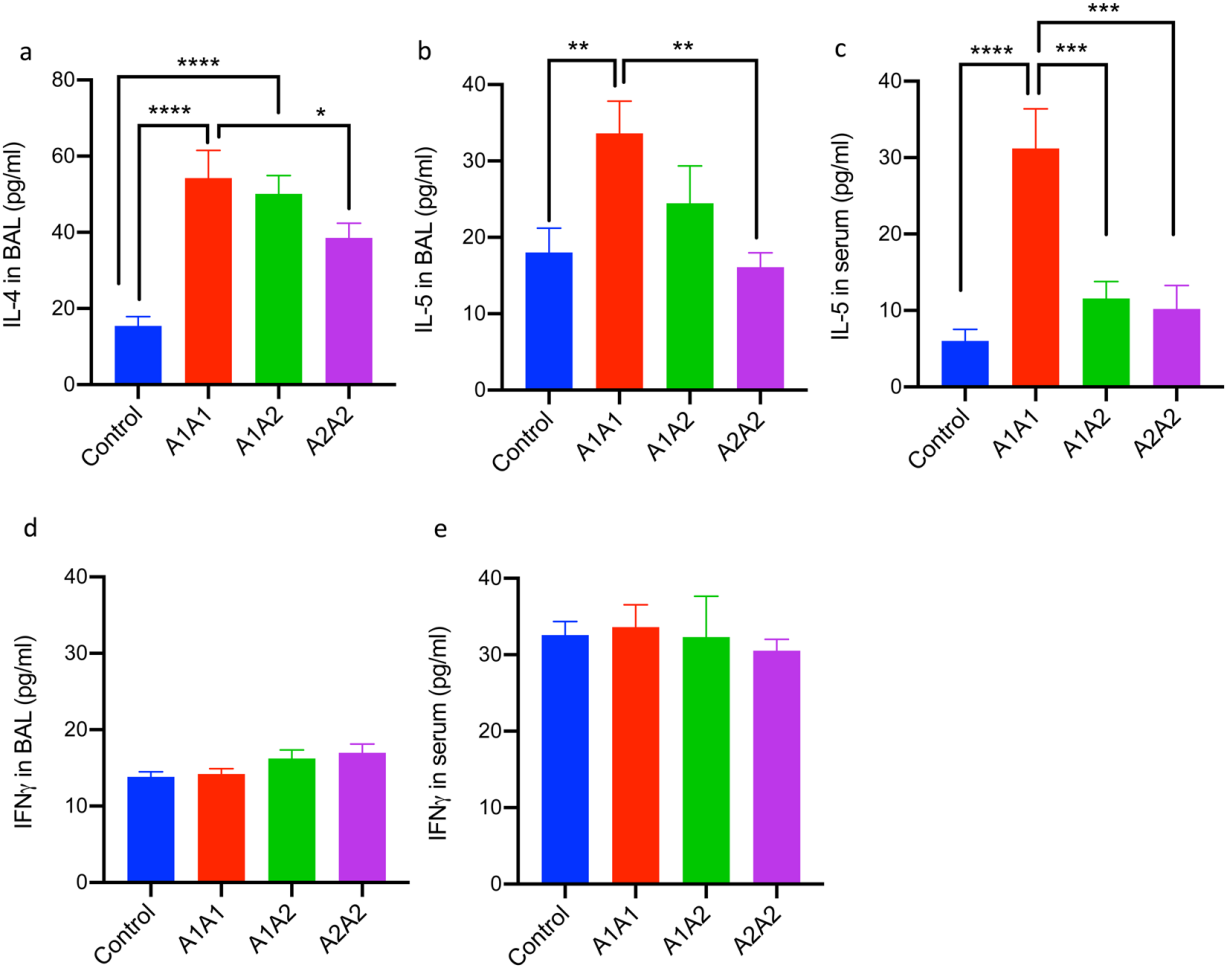
Cytokine profiling after feeding A1A1, A1A2 and A2A2 β casein variants of milk for 30 Weeks.(**a**) IL-4 in BAL, (**b**) IL-5 in BAL, (**c**) IL-5 in serum (**d**) IFN-γ in BAL and (**e)** IFN-γ in serum Results are presented as mean ± standard error of mean (SEM) and analyzed by one-way ANOVA. n = 8–10 mice/group Significance denoted by *P ≤ 0.05, **P ≤ 0.01, ***P ≤ 0.001 and ****P ≤ 0.000.

To evaluate if feeding mice with different β-casein variants of cow milk was resulting in Th1/Th2 imbalance, the levels of representative Th2 (IL-4 and IL-5) and Th1 cytokines (IFN-γ) were measured in BAL as well as in serum (Fig. 2). In BAL, the levels of IL-4 (Fig. 2a) as well as IL-5 (Fig. 2b) were found to be significantly higher in the mice fed with A1A1 milk or A1A2 milk as compared to control or those fed with A2A2 milk. Interestingly, the Th2 cytokine levels in mice fed with A2A2 milk were comparable to the control group and significantly lower than the A1A1 group (Fig. 2a,b). In contrast, no significant difference was observed in the levels of IFN-γ (Fig. 2d). IL-5 concentration in serum of mice fed with A1A1 milk was found to be significantly elevated as compared to control and ones fed with A1A2 and A2A2 milk (Fig. 2c), while IFN-γ levels remained largely unchanged (Fig. 2e).

### Oral feeding of A1A1 β-casein variant of cow milk increases IgG and IgE levels in mice lungs

**Figure 3:**
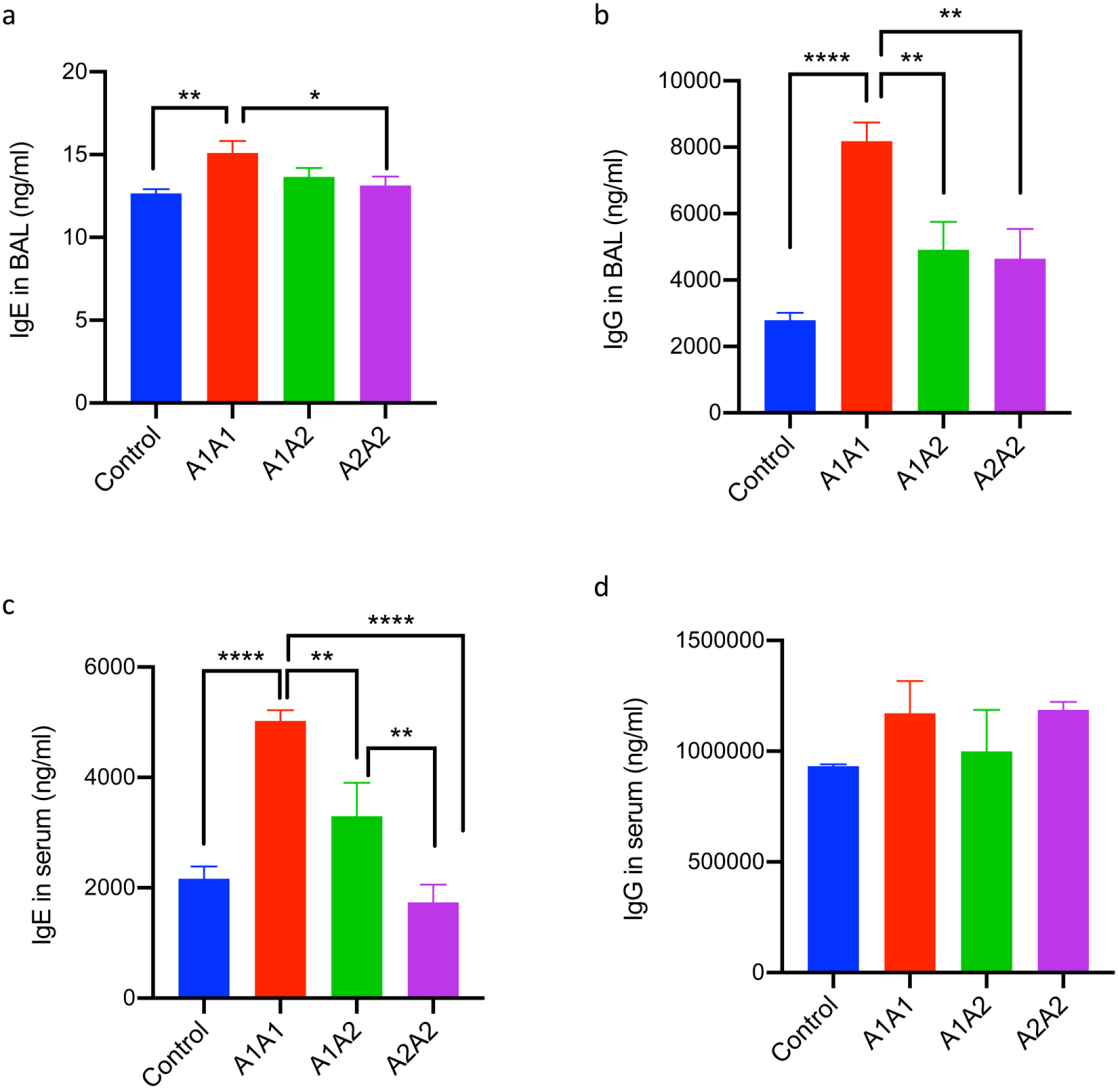
Humoral immune response after feeding A1A1, A1A2 and A2A2 β caesin variants of milk for 30 Weeks. (**a**) IgE in BAL, (**b**) IgG in BAL, (**c)** IgE in serum and (**d**) IgG in serum. Results are presented as mean ± standard error of mean (SEM) and analyzed by one-way ANOVA. n = 6–8 mice/group. Significance denoted by *P ≤ 0.05, **P ≤ 0.01, ***P ≤ 0.001 and ****P ≤ 0.000.

It is known that IL-4-driven immunoglobulin class-switching to IgE plays an important role in hypersensitivity reactions including food allergies and allergic asthma. Hence, we estimated total IgE levels in both BAL and serum (Fig. 3). In the BAL, IgE levels were found to be significantly higher in the mice fed with A1A1 milk as compared to control and A2A2 group (Fig. 3a). Further, there are some studies suggesting a role for allergen-specific IgG in the promotion of Th2-mediated allergic disease in asthma patients. On assessing the same in our study, we found that the IgG levels in BAL were significantly higher in mice fed with A1A1 milk as compared to the control as well as mice fed with A1A2 and A2A2 milk (Fig. 3b). A significant increase in total IgE levels was also observed in the serum of mice fed with A1A1 milk as compared to the A1A2 and A2A2 and control groups. In fact, serum IgE levels in A1A2 milk fed mice were also significantly higher as compared to mice fed with A2A2 milk (Fig. 3c). Of note, serum IgE levels in the mice fed with A2A2 milk were lower than even the control group, though the differences were not statistically significant. No significant differences were observed for serum IgG levels among all the experimental groups (Fig. 3d).

### Increased cellular infiltration of lymphocytes and eosinophils in mice fed with A1A1 β-casein variant of cow milk

**Figure 4:**
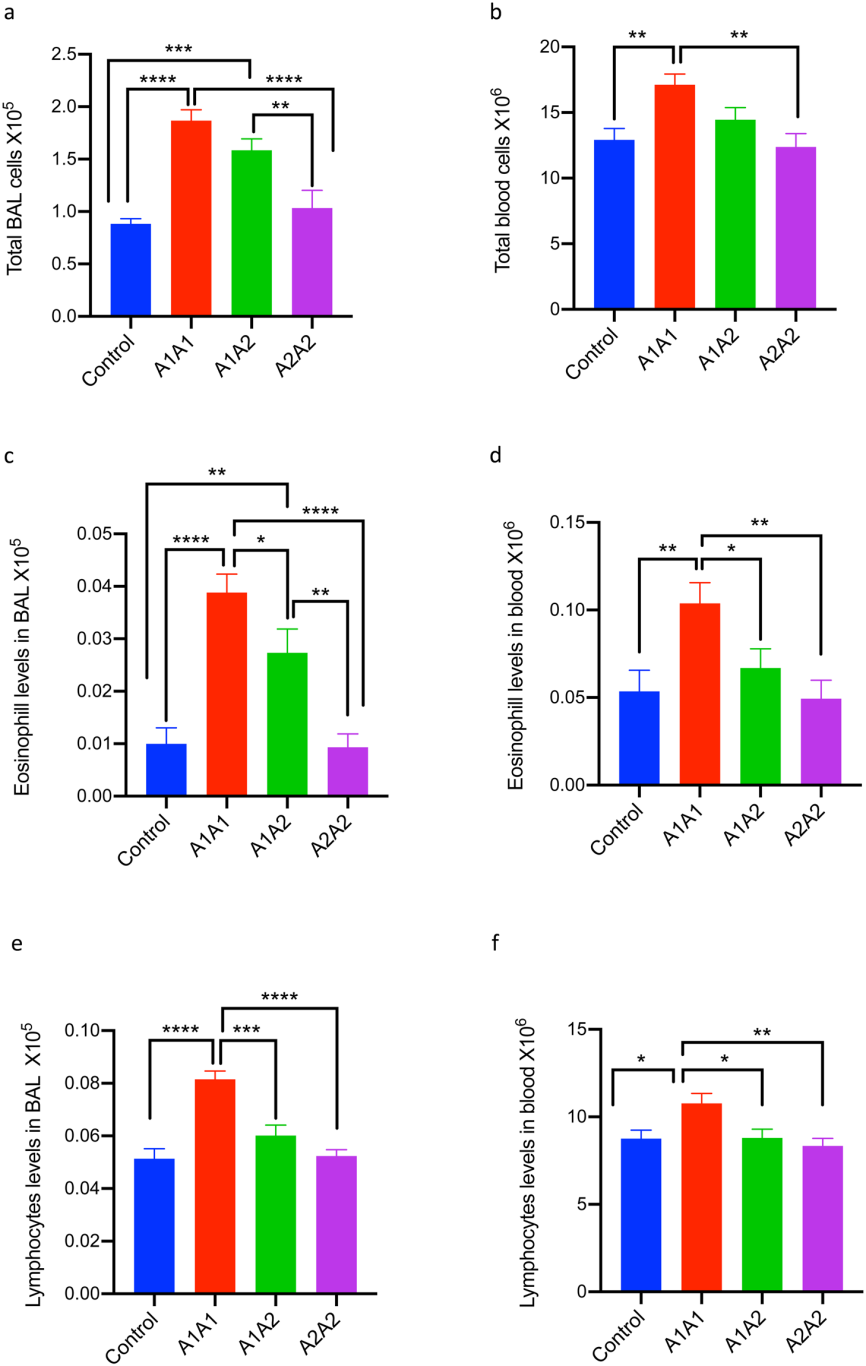
Effect of feeding A1A1,A1A2 and A2A2 β casein variants of milk for 30 Weeks on differential cell counts in bronchoalveolar lavage (BAL) fluid and blood (**a**) Total cells in BAL, (**b**) Total cells in Blood, (**c**) Eosinophils in BAL, (**d**) Eosinophils in blood, (**e)** Lymphocytes in BAL and (**f**) Lymphocytes in blood. Results are presented as mean ± standard error of mean (SEM) and analyzed by one-way ANOVA. n = 6 mice/group. Significance denoted by *P ≤ 0.05, **P ≤ 0.01, ***P ≤ 0.001 and ****P ≤ 0.000.

As inflammation is associated with recruitment of immune cells, we estimated the extent of both pulmonary and systemic inflammation, which was measured by the leukocyte recruitment to the airways and in peripheral blood and was found to be significantly greater in the mice fed with A1A1 milk as compared to those fed with A1A2 and A2A2 milk (Fig. 4**)**. The increase in cellular infiltration in BAL (Fig. 4a) and blood (Fig. 4b) was mainly due to increase in eosinophils (Fig. 4c,d) and lymphocytes (Fig. 4e,f).

### A1A1 β-casein variant of cow milk increases airway inflammation independent of airway remodeling in mice

**Figure 5:**
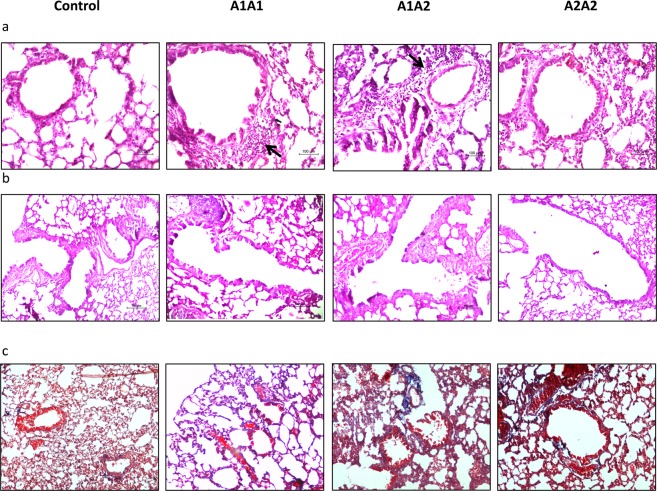
Mouse lungs stained with (**a**) Hematoxylin & Eosin (H&E), (**b**) Periodic acid-Schiff (PAS) and (**c**) Mason Trichrome (MT).The images are representative of 4 animals/group.

Feeding of mice with A1A1 and A1A2 milk led to increase in airway inflammation in lung tissue sections stained with H&E (Fig. 5a), in comparison to control as well as the mice fed with A2A2 milk variant. This result further confirms increased proinflammatory markers in blood and BAL fluid of mice fed with A1A1 milk variant. However, we did not find any difference in mucin (Fig. 5b) and collagen (Fig. 5c) content in the lung sections stained with PAS and MT staining.

## Discussion

Consumption of cow milk in early years of life^21^, especially raw form of milk^22^ has been associated with protective effects for allergies and asthma. A recent study demonstrated that raw cow milk prevented house dust mite-induced airway inflammation in a murine asthma model^16^. However, majority of dairy milk consumed worldwide is processed and it has been established that pasteurization and heat sterilization reduces but does not eliminate BCM7 that is still found at highest levels in milk from cows with A1 variant of β-casein^23^. However, none of these studies have focused on the effects of β-casein variants of cow milk on allergic airway inflammation, unlike compelling evidence from both epidemiological and animal studies implicating the role of A1 β-casein variant in gastrointestinal inflammation^12^.

In present study, mice were fed with A1A1, A1A2 or A2A2 variants of heated cow milk, five days per week with a quantity equivalent to one glass of milk for humans. It has been well established that BALB/c mice develop a good Th2- biased immunological response and have a more robust allergic airway inflammation as compared to other strains like C57BL/6 and this made us chose it as a model for this study. Mice fed with A1A1 milk exhibited heightened AHR, as measured both by Penh and airway resistance, indicating its role in inducing allergy in lungs. The increased levels of Th2 cytokines namely IL-4 and IL-5 in mice fed with the A1A1 variant of cow milk further support this observation. This type of patho-phenotype is typical of Th2 driven allergic asthma^24^. IL-4 is a key cytokine orchestrating pleiotropic changes that occur during allergic responses^25^, including immunoglobulin class-switching from IgG to IgE^26^. IL-5 is another pleiotropic Th2 cytokine that is responsible for eosinophil maturation^27^ and its levels in BAL and serum have been reported to correlate with asthma severity^28^. IgE plays a critical role in both immediate hypersensitivity as well as the late-phase responses characteristic of allergy and asthma. Significantly increased IgE levels found in both BAL and serum of mice fed with the A1A1 milk clearly indicate an immediate hypersensitive response, typical of the allergic asthma phenotype^29^. We also found significantly increased IgG levels in BAL suggesting local pulmonary inflammation. IgG has been found to play a critical role in allergen-specific Th2 inflammation in asthma patients. Studies have shown that allergen-specific IgG generated during primary sensitization complexes with inhaled antigen during secondary responses and depending on the balance of Fc*γ*Rs signalled on DCs, can either positively or negatively affect the development of Th2 inflammation in the lungs^30^. Further, significant increase in total immune cells in both BAL and blood, primarily due to increase in lymphocyte and eosinophil numbers observed in mice fed with A1A1 milk is also in line with an allergic asthma phenotype^29^.

Allergic asthma is a chronic Th2-driven inflammation of the airways and is characterized by bronchial hyperresponsiveness, inflammation of the airways, tissue remodeling, airflow obstruction due to mucus hypersecretion, recruitment of immune cells primarily eosinophils accompanied by increase in IgE levels^24^. Histopathological examination of mouse lungs revealed that while peribronchial inflammation was high in mice fed with A1A1 milk; it was milder in the A1A2 group while no inflammation was observed in mice fed with A2A2 milk. However, no goblet cell hyperplasia and airway remodeling in terms of subepithelial fibrosis was observed even after feeding of milk for a long duration of 30 weeks.

In summary, our results clearly indicate that long-term feeding of A1A1 milk induced significant Th2-driven allergic airway inflammation. However, goblet cell hyperplasia and airway tissue remodeling typical of Th2-driven allergic asthma phenotype^29^ was not observed in the study, perhaps indicating a mild form of the disease phenotype. In future, it will be interesting to study the effects of oral feeding of β-casein variants of cow milk in an allergen-induced mouse model of asthma.

To the best of our knowledge, this is the first study demonstrating the pro-inflammatory effects of dairy milk containing A1 variant of β-casein milk on the respiratory tract. Interestingly, while the A1A1 form was noted to be most inflammatory, A1A2 had intermediate effects and A2A2 milk induced no inflammation but rather seemed to have a protective effect. Our findings are in line with similar studies for gastrointestinal inflammation^12,14^ and also support the notion of cross-talk between the mucosal tissues of our body^31^, though the exact underlying mechanisms need to be better elucidated.

## References

[1] Heck JML (2009). Effects of milk protein variants on the protein composition of bovine milk. J Dairy Sci.

[2] Kamiński S, Cieślińska A, Kostyra E (2007). Polymorphism of bovine beta-casein and its potential effect on human health. J Appl Genet.

[3] Mishra BP (2009). Status of milk protein, â-casein variants among Indian milch animals. Indian J Anim Sci.

[4] Vargas-Bello-Pérez E, Márquez-Hernández RI, Hernández-Castellano LE (2019). Bioactive peptides from milk: animal determinants and their implications in human health. J Dairy Res.

[5] Jinsmaa Y, Yoshikawa M (1999). Enzymatic release of neocasomorphin and β-casomorphin from bovine β-casein. Peptides 1999.

[6] Chia JSJ (2017). A1 beta-casein milk protein and other environmental pre-disposing factors for type 1 diabetes. Nutr Diabetes.

[7] Laugesen M, Elliott R (2003). Ischaemic heart disease, Type 1 diabetes, and cow milk A1 β-casein. Nz Med J Vol..

[8] Truswell AS (2005). The A2 milk case: A critical review. Eur J Clin Nutr.

[9] Singh NK, Kour S, Sharma N (2016). Impact of A1/A2 forms of Cow’s Milk on Human Health-A review. J Anim Res.

[10] Birgisdóttir BE (2002). Influence of nutrition on prevention of diabetes mellitus. Scand J Nutr.

[11] Jarmołowska, B. *et al*. Role of milk-derived opioid peptides and proline dipeptidyl peptidase-4 in autism spectrum disorders. *Nutrients Jan* 4; **11**(1) (2019). 10.3390/nu11010087 PMC6356206 30621149

[12] Brooke-Taylor S, Dwyer K, Woodford K, Kost N (2017). Systematic Review of the Gastrointestinal Effects of A1 Compared with A2 β-Casein. Adv Nutr An Int Rev J.

[13] Jianqin, S. *et al*. Effects of milk containing only A2 beta casein versus milk containing both A1 and A2 beta casein proteins on gastrointestinal physiology, symptoms of discomfort, and cognitive behavior of people with self-reported intolerance to traditional cows’ milk. *Nutr J* **15**, Article number: 35 (2016). 10.1186/s12937-016-0147-z PMC4818854 27039383

[14] Haq MRU, Kapila R, Sharma R, Saliganti V, Kapila S (2014). Comparative evaluation of cow β-casein variants (A1/A2) consumption on Th 2 -mediated inflammatory response in mouse gut. Eur J Nutr.

[15] Perdijk O, Van Splunter M, Savelkoul HFJ, Brugman S, Van Neerven RJJ (2018). Cow’s milk and immune function in the respiratory tract: Potential mechanisms. Front Immunol. Feb 12.

[16] Abbring S (2017). Raw cow’s milk prevents the development of airway inflammation in a murine house dust mite-induced asthma model. Front Immunol Aug 28.

[17] Bartley J, McGlashan SR (2010). Does milk increase mucus production?. Med Hypotheses.

[18] Ganguly I (2013). Beta-casein (CSN2) polymorphism in ongole (Indian Zebu) and frieswal (HF × sahiwal crossbred) cattle. Indian J Biotechnol.

[19] Ram A, Mabalirajan U, Singh SK, Singh VP, Ghosh B (2008). Mepacrine alleviates airway hyperresponsiveness and airway inflammation in a mouse model of asthma. Int Immunopharmacol.

[20] Swaidani S (2013). TSG-6 protein is crucial for the development of pulmonary hyaluronan deposition, eosinophilia, and airway hyperresponsiveness in a murine model of asthma. J Biol Chem.

[21] Riedler J (2001). Exposure to farming in early life and development of asthma and allergy: a cross-sectional survey. Lancet (London, England).

[22] Sozańska B, Pearce N, Dudek K, Cullinan P (2013). Consumption of unpasteurized milk and its effects on atopy and asthma in children and adult inhabitants in rural Poland. Allergy Eur J Allergy Clin Immunol.

[23] Cieślińska A (2012). Milk from cows of different β-casein genotypes as a source of β-casomorphin-7. Int J Food Sci Nutr.

[24] Baos S (2017). Biomarkers associated with disease severity in allergic and nonallergic asthma. Mol Immunol.

[25] Gour N, Wills-Karp M (2015). IL-4 and IL-13 signaling in allergic airway disease. Cytokine.

[26] Poulsen LK, Hummelshoj L (2007). Triggers of IgE class switching and allergy development. Ann Med.

[27] Yanagibashi T, Satoh M, Nagai Y, Koike M, Takatsu K (2017). Allergic diseases: From bench to clinic - Contribution of the discovery of interleukin-5. Cytokine.

[28] Greenfeder S, Umland SP, Cuss FM, Chapman RW, Egan RW (2001). Th2 cytokines and asthma. The role of interleukin-5 in allergic eosinophilic disease. Respir Res.

[29] Schatz M, Rosenwasser L (2014). The allergic asthma phenotype. J allergy Clin Immunol Pract.

[30] Williams JW, Tjota MY, Sperling AI (2012). The contribution of allergen-specific IgG to the development of Th2-mediated airway inflammation. J Allergy (Cairo)..

[31] Tulic MK, Piche T, Verhasselt V (2016). Lung-gut cross-talk: evidence, mechanisms and implications for the mucosal inflammatory diseases. Clin Exp Allergy.

